# Processing of Task-Irrelevant Race Information is Associated with Diminished Cognitive Control in Black and White Individuals

**DOI:** 10.3758/s13415-021-00896-8

**Published:** 2021-05-03

**Authors:** Estée Rubien-Thomas, Nia Berrian, Alessandra Cervera, Binyam Nardos, Alexandra O. Cohen, Ariel Lowrey, Natalie M. Daumeyer, Nicholas P. Camp, Brent L. Hughes, Jennifer L. Eberhardt, Kim A. Taylor-Thompson, Damien A. Fair, Jennifer A. Richeson, B. J. Casey

**Affiliations:** 1 Department of Psychology, Yale University, Estée Rubien-Thomas, 2 Hillhouse Ave, New Haven, CT 06511 USA; 2 Columbia University College of Physicians and Surgeons, New York, NY USA; 3 Department of Behavioral Neuroscience, Oregon Health and Science University, Portland, OR USA; 4 Department of Psychology and Neural Science, New York University, New York, NY USA; 5 Department of Organizational Studies, University of Michigan, Ann Arbor, MI USA; 6 Department of Psychology, University of California Riverside, Riverside, CA USA; 7 Department of Psychology, Stanford University, Stanford, CA USA; 8 School of Law, New York University, New York, NY USA; 9 Masonic Institute for the Developing Brain, Minneapolis, MN USA

**Keywords:** Cognitive control, Race, Attention, fMRI, Face perception, Implicit bias

## Abstract

The race of an individual is a salient physical feature that is rapidly processed by the brain and can bias our perceptions of others. How the race of others explicitly impacts our actions toward them during intergroup contexts is not well understood. In the current study, we examined how task-irrelevant race information influences cognitive control in a go/no-go task in a community sample of Black (n = 54) and White (n = 51) participants. We examined the neural correlates of behavioral effects using functional magnetic resonance imaging and explored the influence of implicit racial attitudes on brain-behavior associations. Both Black and White participants showed more cognitive control failures, as indexed by dprime, to Black versus White faces, despite the irrelevance of race to the task demands. This behavioral pattern was paralleled by greater activity to Black faces in the fusiform face area, implicated in processing face and in-group information, and lateral orbitofrontal cortex, associated with resolving stimulus-response conflict. Exploratory brain-behavior associations suggest different patterns in Black and White individuals. Black participants exhibited a negative association between fusiform activity and response time during impulsive errors to Black faces, whereas White participants showed a positive association between lateral OFC activity and cognitive control performance to Black faces when accounting for implicit racial associations. Together our findings propose that attention to race information is associated with diminished cognitive control that may be driven by different mechanisms for Black and White individuals.

**Supplementary Information:**

The online version contains supplementary material available at 10.3758/s13415-021-00896-8.

Day-to-day life often involves interactions with people from social groups different from our own. Group membership can affect how we view (Walker & Hewstone, 2006), evaluate (Perdue et al., 1990), and behave toward others (Tajfel et al., 1971). Race remains a salient indicator of who we consider an in-group member. A large body of work has addressed how we perceive and attend to individuals from other racial groups. However, less is known about the effects of race information on explicit actions in interracial contexts. The current study examined cognitive control in the presence of task-irrelevant race information (Black and White faces) and investigated the underlying neural correlates in Black and White participants. Specifically, we examined associations between sensitivity to race information in early visual and prefrontal control circuitry, cognitive control performance, and racial group membership. Understanding the cognitive and neural processing of task-irrelevant race information and its influence on actions may help to elucidate disparate behaviors during interracial encounters.

The significance of race traces back to evolutionary origins and the need to identify coalitional alliances for survival (e.g., members of our in-group versus out-group; (Cosmides et al., 2003). In the United States, where this study was conducted, the social construct of race remains a malleable (Richeson & Sommers, 2016), yet salient (Taylor et al., 1978) heuristic for group membership, where factors such as physical features (Maddox & Gray, 2002), stereotyping (Hugenberg & Bodenhausen, 2004), and societal contexts (Rodeheffer et al., 2012) contribute to race categorization.

## Attentional bias in the presence of race information

The visual salience of race information can have instrumental effects on one’s ability to impose control on behavior. Cognitive control is the ability to suppress inappropriate thoughts, emotions, and actions in favor of goal-oriented ones (Casey, 2015; Miller & Cohen, 2001). It is thought to be vital to successfully negotiating diverse emotional and social contexts (Cohen et al., 2016; Steinbeis et al., 2012) that require redirecting attention or withholding inappropriate responses. Research conducted in predominantly White samples shows that Black faces capture attention more quickly than White faces (Bean et al., 2012; Donders et al., 2008; Ito & Urland, 2003; Trawalter et al., 2008) and prime the detection of stereotype-congruent objects (e.g., guns indicating danger; Eberhardt et al., 2004; Payne, 2001; Payne et al., 2002; Todd et al., 2016).

Attentional bias to Black faces is often interpreted as reflecting the activation of stereotypes of Black individuals as physically threatening or dangerous (Cottrell & Neuberg, 2005; Duncan, 1976; Gilbert, 1951; Karlins et al., 1969; Katz & Braly, 1933; Kleider-Offutt et al., 2018; Shapiro et al., 2009; Skinner & Haas, 2016). These findings mirror the attentional bias literature on responses to biological threats (Ohman et al., 2001), suggesting that Black faces are rapidly encoded as threatening stimuli among White individuals. Recent empirical and theoretical work suggests that the effects of racial stereotypes and biased attention to race can extend to the processing of other social categories, such as gender and age (Freeman et al., 2011; Goff et al., 2014; Johnson et al., 2012; Stolier & Freeman, 2016), exemplifying the wide-ranging effects of this phenomenon.

Evidence from brain imaging studies supports the rapid processing of and attentional bias to race information. Experiments using event-related potentials demonstrate that the brain processes race and out-group information early and rapidly (Amodio, 2014; Walker et al., 2008), before the processing of other salient social categories, such as gender (Ito & Urland, 2003; Ito & Urland, 2005). The rapid processing of race occurs even when race information is irrelevant to current goals, suggesting that the encoding of race often occurs in a bottom-up, automatic fashion (Colombatto & McCarthy, 2017; Ito & Urland, 2003; Kubota & Ito, 2007, 2017). Functional neuroimaging (fMRI) studies examining the processing of race have focused in part on the Fusiform Face Area (FFA), a brain region associated with early and rapid visual processing of faces (Kanwisher et al., 1997; McCarthy et al., 1997) and social categories (Van Bavel et al., 2008). Several studies provide evidence that the FFA distinguishes between same- versus other-race faces in both the magnitude and spatial representation of neural activity (Brosch et al., 2013; Contreras et al., 2013; Golby et al., 2001; Hughes et al., 2019; Kaul et al., 2014; Levin & Banaji, 2006; Natu et al., 2011; Ofan et al., 2011, 2014; Ratner et al., 2013; Reggev et al., 2020). This work demonstrates that FFA activity reflects rapid engagement of visual and attentional processes in response to race information and provides reason to hypothesize its role in cognitive control performance in the presence of task-irrelevant race information.

## Cognitive control in the presence of race information

Numerous studies have demonstrated altered cognitive control performance in response to Black cues (Bartholow et al., 2006; Correll et al., 2002, 2006; Donders et al., 2008; Richeson et al., 2003; Richeson & Shelton, 2003; Senholzi et al., 2015), which is paralleled by altered engagement of prefrontal circuitry (Amodio et al., 2004; Brown et al., 2017; Richeson et al., 2003). Even simple exposure to racial out-group members activates prefrontal control regions (Cunningham et al., 2004; Lieberman et al., 2005; Richeson et al., 2003), suggesting that the presence of Black faces may engage cognitive control related processes related to threat or concern by subjects of appearing prejudiced (Amodio, 2014). The interpretation of Black faces precipitating stress is in accordance with findings demonstrating that threat manipulations alter prefrontal connectivity and function (Arnsten, 2015; Lindström & Bohlin, 2012; Liston et al., 2009). As a whole, this research work supports the notion that race information can direct attention to task-irrelevant physical features and interfere with goal-oriented cognitive processes via changes in prefrontal circuitry and function.

Traditionally, orbitofrontal cortex has been implicated in response inhibition tasks (Elliott et al., 2000). More recent work associates the orbitofrontal cortex with attentional control (Vuilleumier et al., 2001) and resolving stimulus-response conflict (Mansouri et al., 2014), both components of cognitive control processes. The lateral orbitofrontal cortex (lOFC) plays an important role in the dissociation of responses to stimuli (Bryden & Roesch, 2015) and the suppression of unwanted responses (Iversen & Mishkin, 1970) required for successful cognitive control. The lOFC’s role in resolving response conflict suggests its potential involvement in executing cognitive control in the presence of race information.

Individual and social factors associated with racial group membership, such as implicit racial attitudes (Nosek & Greenwald, 2002), allow for further granularity in our understanding of the processes that underlie biased behavior in the presence of task-irrelevant race information. Several studies have shown that activity in early face processing regions in response to race cues is associated with pre-existing implicit racial attitudes (Brosch et al., 2013; Ofan et al., 2011). Associations with implicit attitudes extend to higher-order cognition, where implicit racial attitudes moderate brain activity and cognitive control performance in the presence of race information (Brosch et al., 2013; Phelps et al., 2000; Richeson et al., 2003, 2005). Consideration of additional factors that may contribute to biased behavior is crucial for constraining interpretations that often represent the oversimplification of complex processes.

## Current Study

Although past research has examined perceptual and cognitive processes to out-group faces in passive viewing tasks (Cunningham et al., 2004; Lieberman et al., 2005; Richeson et al., 2003, 2005), categorization paradigms (Cassidy & Krendl, 2016, 2016; Ratner et al., 2013; Ronquillo et al., 2007; Stolier & Freeman, 2016; Van Bavel et al., 2008), and in threatening contexts (Correll et al., 2002), less work has examined whether task-embedded race information directly diminishes cognitive control to cues in more benign contexts. Furthermore, many of the relevant neuroimaging studies to date have included relatively small samples and/or predominately non-black samples (Brown et al., 2017; Hart et al., 2000; Mathur et al., 2012), which preclude differentiation of effects driven by race of stimuli (e.g., all individuals regardless of their race showing a bias toward Black faces) versus out-group membership (i.e., White individuals show biased behavior to Black faces and Black individuals show biased behavior to White faces). The primary goal of this study was to examine the cognitive and neural processes associated with behavioral performance to same- versus other-race faces during a cognitively demanding task in both Black and White participants. Based on the existent literature, we hypothesized that: 1) both Black and White individuals would show lapses in cognitive control performance in response to Black faces (Brown et al., 2017; Correll et al., 2002; Richeson et al., 2003); and 2) Black and White individuals would show engagement of visual processing regions associated with face and race processing (e.g., FFA; Brown et al., 2017; Ofan et al., 2011), and lateral prefrontal circuitry previously implicated in cognitive control (Brown et al., 2017; Cassidy & Krendl, 2016; Gilbert et al., 2012; Richeson et al., 2003). To constrain the interpretations of our findings, we performed exploratory brain-behavior analyses and probed whether implicit racial attitudes, as measured by the Implicit Association Test (Greenwald et al., 1998), contributed to our observed associations in distinct or similar ways based on racial group membership (e.g., effects driven by race information or by out-group relation).

## Methods

### Sample

A community sample of 106 Black and White, healthy right-handed adults, 18-37 years (mean age = 26.08, SD = 5.21, 55 Black, 57 females) were recruited from the greater New York City and New Haven, Connecticut metropolitan areas. One participant (Black male) was excluded for technical difficulties in the scanner, leaving a total of 105 participants with imaging and behavioral data (Figure S1). None of the participants reported previous or current diagnoses of psychiatric or neurological disorders, or the use of psychotropic medications. All participants provided written consent approved by institutional review boards at their respective data collection sites.

### Behavioral Paradigm

**Fig. 1:**
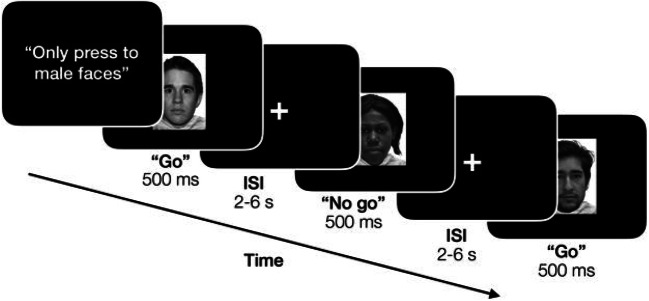
Experimental go/no-go paradigm. The example run illustrates the condition of male faces as targets (go trials) and female faces as nontargets (no-go trials). Blocks of trials for which the target was a male or a female face were presented in a randomized order for each participant

The go/no-go task consisted of male and female faces displaying a neutral expression that served as both targets and nontargets (Somerville et al., 2011; Figure 1). Participants were instructed to press a button as quickly as possible for targets and withhold a response for nontargets. At the beginning of each run, participants were told to detect either male or female faces; thus, target race was orthogonal to the instructed task. Within each trial, a face appeared for 500 milliseconds, followed by a jittered intertrial interval (2-6 seconds). A total of 102 trials were presented in a pseudo-randomized order within each run (72 go trials, 30 no-go trials). The ratio of go versus no-go trials was chosen to create a pre-potent bias to respond across trials to maximize false-alarm rates (Young et al., 2018). Data were acquired in two 7-min 3-sec runs, consisting of both combinations of male and female faces as either the targets or nontargets. The order of runs was randomized across subjects.

To test for differences in responses to Black versus White faces, the task was optimized to include 45% White faces, 45% Black faces, and 10% “foil” faces (Asian and Hispanic, to keep participants naïve to study hypotheses), which were equally distributed across each experimental condition. Stimuli harmonized for luminosity, head size, and head position were obtained from two previously validated sets of face stimuli (Conley et al., 2018; Tottenham et al., 2009). All stimuli displayed “calm” faces and had emotional expression validity ratings of 0.7 or higher. Lastly, participants completed a well-validated measure of implicit racial attitudes (IAT, Greenwald et al., 1998) in which individuals sorted photographs of faces of Black and White Americans and positive and negative evaluative words into stereotype-congruent (Black/bad & White/good) and stereotype-incongruent (Black/good & White/bad) blocks of trials.

### fMRI Data Acquisition

Sequence parameters were based on previously published ABCD imaging parameters (Casey et al., 2018). Images were acquired on two Siemens Prisma 3 T scanners (version VE11B). Anatomical images were acquired using a contrast between gray and White matter using T1-weighted sequence (repetition time (TR) = 2,400 ms; echo time (TE) = 2.12 ms; T1 = 1,060 ms; flip angle, 8 degrees; 256 × 256 matrix; sagittal slices, 208; resolution, 1 mm^3^) and T2-weighted images (repetition time (TR) = 3,200 ms; echo time (TE) = 564 ms; 256 × 256 matrix; sagittal slices, 208; resolution, 1 mm^3^). Whole-brain echo-planar imaging (EPI) volumes were acquired with T2*-weighted EPI sequence sensitive to the blood-oxygenation-level-dependent (BOLD) contrast (TR = 800 ms; TE = 30.0 ms; flip angle, 49 degrees; voxel size 2.4 mm^3^; AC-PC oriented slices, 66). MRI data were acquired using the same imaging parameters at both sites.

### Behavioral Data Analysis

All statistical analyses were conducted using R (R Core Team, 2018). Dprime, a sensitivity index comprising hit rates (correct go trials) and false-alarm rates (incorrect no-go trials) was the primary dependent measure of cognitive control (Macmillan & Creelman, 2004). Instances of optimum hit rates were represented similarly across stimulus race and participant race (Figures S2A and S2B), indicating that dprime scores in the present sample represent a sensitivity index rather than of systematic differences in miss rates between Black and White stimuli. Adjusted dprime scores were calculated with a log-linear approach (Hautus, 1995) which corrects for extreme values in hit and false-alarm rates. Response bias scores were calculated using *c* (Banks, 1970; Ingham, 1970), a measure of decision criteria that is independent from dprime (Stanislaw & Todorov, 1999). Signal detection and response bias scores were obtained using the *dprime* function in the “Psycho” package. Summary statistics for within-subject designs were computed based on Morey (2008) by using the *SummarySEWithin* function in the “Rmisc” package.

In our primary behavioral analysis, a linear mixed effect model was used to determine the effects of stimulus race on dprime scores. Mean-centered dprime scores were submitted to analyses, so that y-intercepts reflected the grand mean of the dependent variable (Afshartous & Preston, 2011). Between-subject factors of participant race and participant gender, and within-subject factors of stimulus race and stimulus gender race were treated as fixed effects with interaction terms between primary factors of interest (participant race, stimulus race, and stimulus gender). A secondary linear mixed effects analysis was conducted to model the effects participant race and stimulus race, and trial type (go vs. no-go trials) on accuracy. The “lme4” package (Bates et al., 2015) was used to perform linear mixed effects analyses, where subjects were treated as a random effect nested within site (i.e., random intercepts for each participant). Fixed factors for both linear mixed effect analyses were represented with deviation coding, allowing for interpretation of interaction effects. Visual inspection of all residual plots revealed normal distributions for each model with no obvious deviations from homoscedasticity or normality. Full models were compared to the appropriate null model using likelihood ratio tests.

Scoring of the Implicit Association Test followed the algorithm recommended by Greenwald and Nosek (2003), where D-scores were calculated for each participant as a standardized measure of the relative strength of positive and negative associations with Black faces. Positive scores indicate pro-white attitudes and negative scores indicate pro-black attitudes. Six participants (4 Black, 2 White) had invalid scores (>10% of trials had response times <300 ms) based on the Greenwald and Nosek (2003) scoring algorithm.

### fMRI Data Analysis

Structural and functional imaging data were preprocessed using the Human Connectome Project (HCP) Minimal Preprocessing Pipeline version 3.17 for image correction, localization, and registration (described in detail in Glasser et al., 2013). Specifically, EPI images were corrected for gradient distortions using spin echo field maps with opposite phase encoding directions, intensity normalized to the grand-mean, corrected for head motion, and transformed to standard space (MNI 152, 2mm voxels) using nonlinear registration. Volumetric outputs from the fMRI processing step in the HCP Minimal Preprocessing Pipeline were used for analyses. FSL [FMRIB Software Library, (Woolrich et al., 2001)] was used for neuroimaging analyses.

Twelve first-level regressors were created for each participant within the task: Black Correct Go trials, White Go Correct trials, Foil Correct Go trials, Black Correct No-go trials, White Correct No-go trials, Foil Correct No-go trials, Black Incorrect Go trials, White Go Incorrect trials, Foil Incorrect Go trials, Black Incorrect No-go trials, White Incorrect No-go trials, and Foil Incorrect No-go trials. Stimulus gender was omitted from first-level regressors based on lack of interdependence between stimulus race and stimulus gender in the dprime analysis. Generation of the 12 original regressors allowed for analysis of contrasts of interest based on stimulus race, trial type, and performance for each individual subject using first-level FEAT (Woolrich et al., 2001). Each trial was modeled for 500 milliseconds, corresponding to the duration of the stimulus presentation, and convolved with a double-gamma hemodynamic response function. Temporal derivatives of each stimulus predictor were included as additional regressors. Time series data were pre-whitened to account for autocorrelations. Regressors modeling six movement parameters were included in the general linear model (GLM) for each participant. Volumes with greater than 0.9-mm frame-wise displacement were excluded from analyses, as well the neighboring volume before and after (Power et al., 2014). First-level analyses were smoothed at 5-mm full width, half maximum of the Gaussian kernel. Second-level analyses calculated mean response across runs of the task for each participant. Mixed-effect group level analyses were performed using FSL’s Group-level FEAT (Woolrich et al., 2004). All group level analyses included between-subjects factors of participant race, participant gender, and scanning site. Group-level analyses Z-statistic images were thresholded by using Gaussian Random Field theory with a corrected cluster significance threshold of Z > 3.1 and cluster significance threshold of *p* < 0.05 (Worsley, 2001).

Exploratory analyses were conducted to examine associations between the BOLD signal in identified regions of interest (ROIs) defined from whole brain analyses and task performance. Cognitive control failures were measured by dprime, which incorporates both false alarm and hit rates, and by speed of impulsive errors (i.e., false-alarm reaction times [RTs]). IAT D scores were included as a covariate to account for the impact of racial implicit attitudes in any associations. Percent signal change in ROIs was extracted for each subject using FSL’s *FeatQuery* function. Multiple linear regressions were conducted using percent signal change in ROIs from contrasts of interest and participant race to predict differences in cognitive performance in response to Black and White faces. Simple slopes analyses were examined for each participant group. Post-hoc analyses were conducted in R (R Core Team, 2018).

## Results

### Behavioral Results

**Fig. 2:**
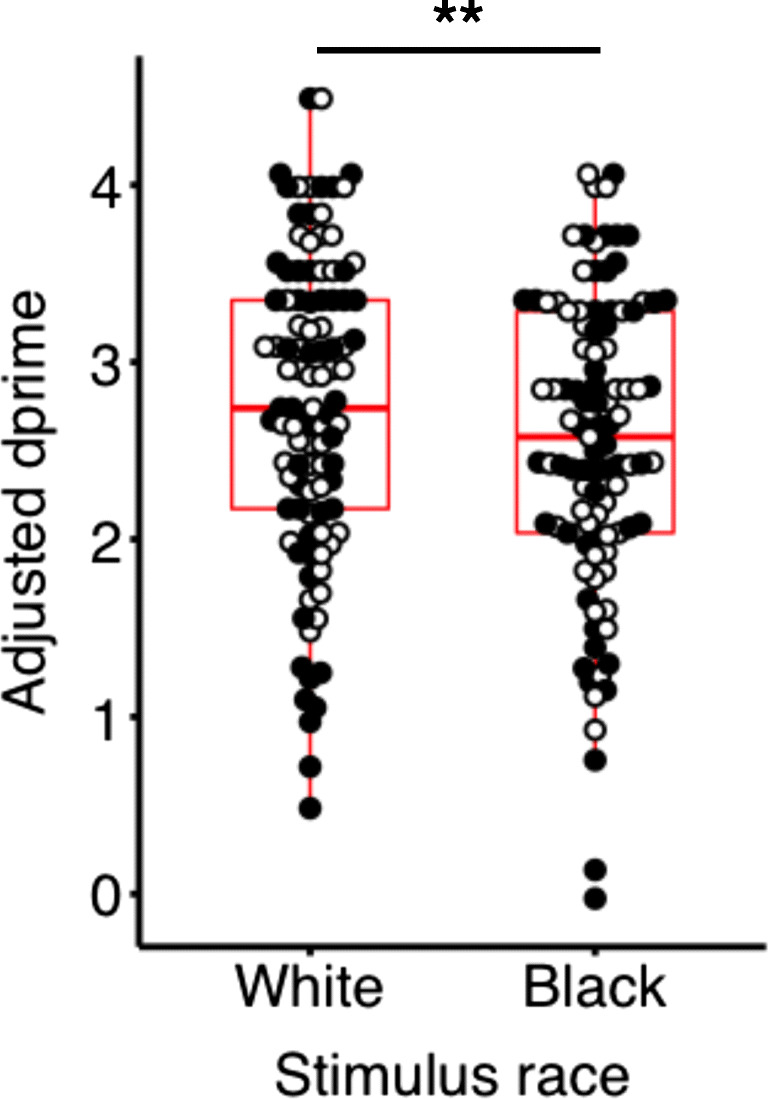
Both Black and White participants show poorer cognitive control to Black versus White faces. Boxplots represent the first and third quartile, median, and range of adjusted dprime scores. Dprime scores for White and Black participants are represented by White and Black dots, respectively

Participants demonstrated a high ratio of hit trials to false-alarm trials, indicating that participants were attending to the task (Figure S2A). Response bias scores were similar across participant and stimulus race (Figure S2B) and were within the expected range considering the number of trials, intertrial intervals, and percent no-go trials in the paradigm (Young et al., 2018). Consistent with Hypothesis 1, a linear mixed effect model revealed a significant effect of stimulus race on task performance. Regardless of participant race, adjusted dprime scores to Black faces (M = 2.55, SD = 0.84) were lower than to White faces (M = 2.76, SD = 0.86; *F*(1, 311.00 = 7.40, *p* = 0.0069; Fig. 2). A main effect of stimulus gender was also observed (female trials: M = 2.50, SD = 0.99, male trials: M = 2.91, SD = 0.84; *F*(1, 311.00 = 47.72, *p* < 0.001; Figure S5A) also was observed. There were no interactions between the primary independent variable of stimulus race with stimulus gender or participant race (stimulus race x stimulus gender: *p* = 0.61; stimulus race x participant race: *p* = 0.64; Table S1). Akaike information criterion (AIC) was used as an index of the statistical quality of the model (Akaike, 1973). Likelihood ratio testing comparing the full model against a model without the fixed effect of interest (stimulus race) revealed a significant difference (*p* = 0.050), with the full model minimizing information criteria to a greater extent (AIC = 1063.0, R_marginal_^2^ = 0.079) than the null model (AIC = 1064.9, R_marginal_^2^ = 0.072).

The secondary behavioral analysis predicting accuracy from participant race, stimulus race, and trial type revealed a significant interaction between participant race and trial type (*F*(1,309) = 6.56, *p* = 0.011) and a trending level interaction between stimulus race and trial type (*F*(1,309) = 3.00, *p* = 0.084. Because it is directly relevant to the study’s hypothesis, the stimulus race by trial type interaction was examined further. While there was no significant difference between the White (M = 0.95, SD = 0.078) and Black (M = 0.95, SD = 0.078, *p* = 0.72) go trials, a significant difference in accuracy to no-go trials was observed, where accuracy was lower in response to Black faces (M = 0.75, SD = 0.19) compared with White faces (M = 0.79, SD = 0.17, *p* < 0.001). Likelihood ratio testing comparing the full model against a model without stimulus race by trial type interaction revealed a significant difference (*p* = 0.022) where the full model minimized information criteria (AIC = −491.4, R_marginal_^2^ = 0.30) to a greater extent than the null model (AIC = −487.7, R_marginal_^2^ = 0.29), suggesting that diminished performance to Black faces may be driven primarily by inaccuracy in no-go trials.

### Imaging Results

#### Cognitive control effects

Correct no-go versus correct go trials exhibited increased activity in bilateral inferior frontal gyrus (Z_max_ = 5.25, *p* < 0.05 cluster corrected), medial prefrontal cortex (Z_max_ = 4.70, *p* < 0.05 cluster corrected), and bilateral parietal cortex (Z_max_ = 5.35, *p* < 0.05 cluster corrected; Figure S4; Table S2). Robust motor activity was observed for the contrast of correct go versus correct no-go trials in our right-handed sample in left precentral gyrus (Z_max_ = 10.3, *p* < 0.05 cluster corrected) and right cerebellum (Z_max_ = 10.2, *p* < 0.05 cluster corrected; Figure S4, Table S3).

#### Stimulus race effects

**Fig. 3:**
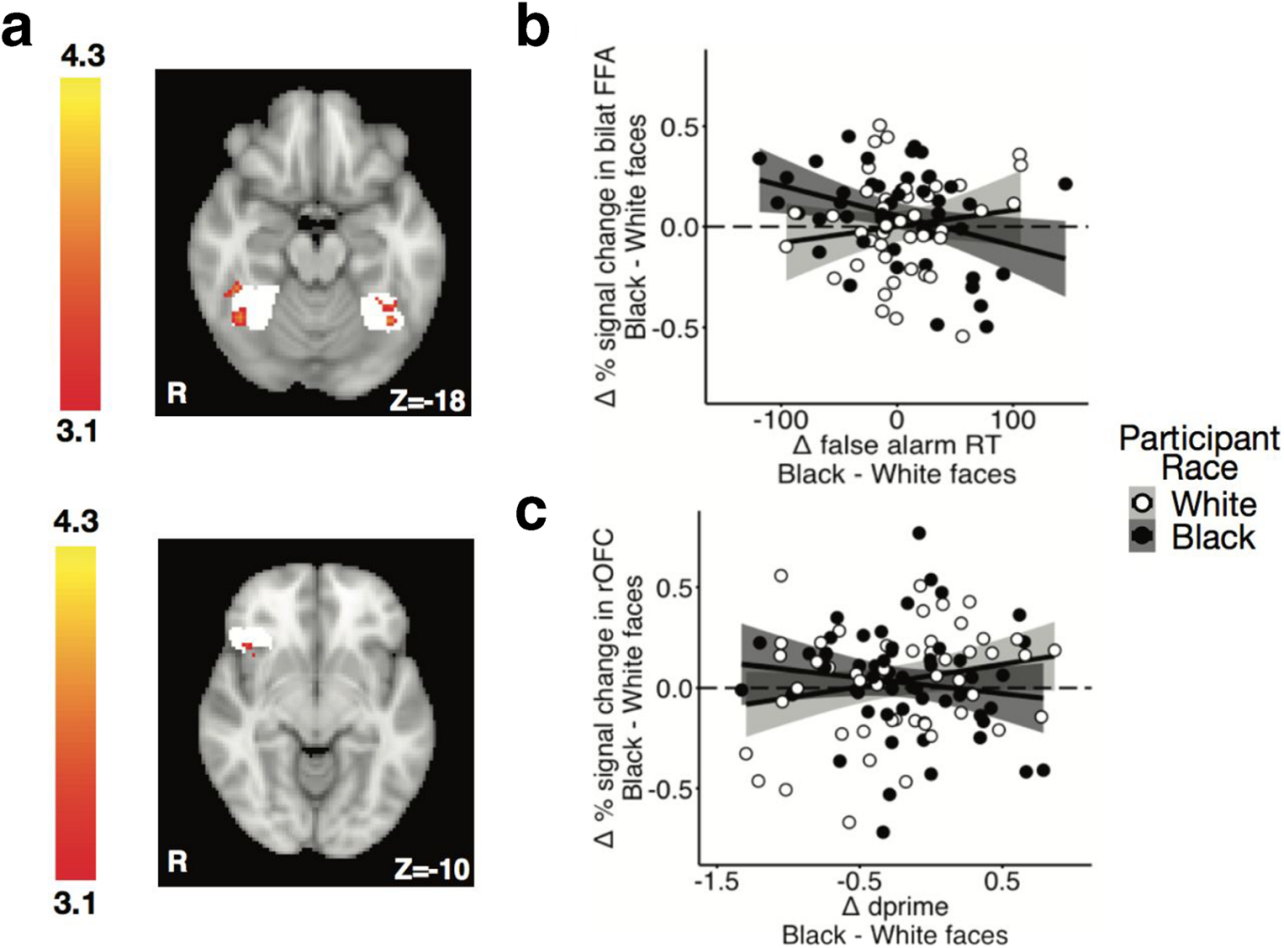
Stimulus race associations with behavior and brain activity. **A)** Individuals, regardless of their own race, show enhanced BOLD signal in response to Black versus White faces in bilateral fusiform and right lOFC. Color bar represents Z-scores. White underlay represents anatomical ROIs. **B**) Black participants show a negative association between activity in bilateral FFA and false alarm RT to Black compared with White faces. No association was observed in White participants. **C**) In contrast, White participants show that greater right lOFC activity is associated with better performance (as measured by dprime) to Black versus White faces when controlling for implicit racial associations. No association was observed for Black participants. lOFC = lateral orbitofrontal cortex; FFA = fusiform face area; RT = reaction time

Given the observed diminished cognitive control to Black versus White faces by both Black and White participants, we tested for differences in the bold signal to all Black relative to all White faces using a whole brain contrast. Participants showed greater BOLD responses bilaterally in the fusiform (Z_max_ = 4.24, *p* < 0.05 corrected) and in the right lateral orbitofrontal cortex (right lOFC; Z_max_ = 4.08, *p* < 0.05 corrected) to Black relative to White faces (Figure 3A; Table S4). A post-hoc analysis comparing the two primary contrasts of interest revealed an interaction between participant race and stimulus race in the ventromedial PFC (Z_max_ = 6.05, *p* < 0.05 cluster corrected), bilateral parietal cortex (Z_max_ = 5.75, *p* < 0.05 cluster corrected), and precuneus (Z_max_ = 4.70, *p* < 0.05 cluster corrected), where Black participants showed more activation than White participants in the Black versus White faces contrast for all three regions (Table S5).

#### Exploratory Analysis of Brain-Behavior Associations

To constrain the interpretation of our findings of greater fusiform and orbitofrontal activity to Black versus White faces, we examined BOLD responses in these regions in relation to participant race, and frequency and speed of cognitive control failures as measured by dprime and false alarm reaction times (RTs). Models of decision-making under time constraints provide evidence that signal detection and reaction time measures represent two distinct yet related components of decision making processes (Ratcliff et al., 2016; Swensson, 1972). The exploratory brain-behavior analyses were performed to allow for the differentiation between stimulus-race effects (i.e., Black and White subjects show similar brain-behavior patterns) versus out-group effects (i.e., Black and White subjects show distinct brain-behavior patterns) in any observed associations. Percent signal change in the Black versus White faces contrast was extracted from neuroanatomically defined ROIs in “bilateral temporal fusiform cortex” and “right frontal orbital cortex” using the Harvard-Oxford atlas (Desikan et al., 2006).

First, given the association of the FFA with early and rapid visual processing of face, in-group and race information (Brosch et al., 2013; Golby et al., 2001; Hughes et al., 2019; Kanwisher et al., 1997; Kaul et al., 2014; Levin & Banaji, 2006; McCarthy et al., 1997; Natu et al., 2011; Ofan et al., 2011, 2014; Ratner et al., 2013), we tested for a possible association between BOLD activity in the FFA and a measure that captures the influence of rapid perceptual processing of race information on cognitive control failures. Reaction times reflect visual attentional biases and can be modulated by race information (Eberhardt et al., 2004), providing reason to examine response times in false-alarm trials where stimulus race effects may interfere with the desired response to gender cues. We tested for an association between BOLD activity in the bilateral fusiform and speed of impulsive errors to Black versus White faces (i.e., false-alarm RT to Black faces – false-alarm RT to White faces). To determine specificity of this association to perceptual processing speed, we also tested for an association between FFA activity and the overall measure of cognitive control performance for Black relative to White faces. We observed an interaction between participant race and BOLD activity to stimulus race in predicting differential false-alarm RTs (B= 107.26, *t*(85) = −2.49, *p* = 0.03 Benjamini-Hochberg corrected, *f*^*2*^ = 0.07, 95% CI [0.0, 0.23]; Figure 3B).^1^^,^^2^ Simple slopes analyses revealed an association for Black participants where greater fusiform activity was associated with faster impulsive errors to Black faces (B = −81.24, *t*(85) = −2.65, *p* = 0.0097) that was not observed for White participants (*p* = 0.39). The observed interaction showed marginal significance when including IAT scores as a covariate in the model (B = −102.15, *t*(79) = −2.22, *p* = 0.06 Benjamini-Hochberg corrected, *f*^*2*^ = 0.06, 95% CI [0.0, 0.22]), where Black participants with greater pro-black attitudes appearing to contribute most to this association (Figure S6A). No associations were observed between participant race and BOLD activity in bilateral FFA in predicting adjusted dprime scores, demonstrating specificity of the above brain-behavior association to the fusiform area (*p*s > 0.30 corrected).

Second, we tested whether right lOFC activity predicted differential dprime scores (i.e., dprime to Black faces – dprime to White faces). The lOFC has been implicated in processes critical for cognitive control (e.g., response inhibition), motivating our exploratory analysis of differential BOLD activity in the lOFC and cognitive control performance to Black and White faces. Lesion (Iversen & Mishkin, 1970) and neuroimaging (Chikazoe et al., 2009) studies have demonstrated that the lOFC is critical for response inhibition and that enhanced OFC activity is involved in suppressing unwanted responses (Iversen & Mishkin, 1970). Relatedly, nonhuman primate research implicates the lOFC in dissociating between similar actions during stimulus-response conflict (Bryden & Roesch, 2015). Dprime, a measure of signal detection, provides an index of how well perceptually driven actions are dissociated. As such, we tested whether lOFC activity predicted dprime scores to Black faces relative to White faces. No interaction was observed between signal change and participant race in predicting adjusted dprime scores (*t*(100) = −1.98, *p* = 0.10 Benjamini-Hochberg corrected, *f*^*2*^ < 0.001, 95% CI [0.0, 0.06]).^3^ When including IAT score as a covariate in the model, the observed reaction was significant (*t*(93) = −2.47, *p* = 0.030 Benjamini-Hochberg-corrected *f*^2^ = 0.07, 95% CI [0.0, 0.21]). Greater BOLD response in right lOFC to Black versus White faces marginally predicted higher dprime scores to Black faces in White participants (B = 0.46, *t*(100) = 1.74, *p* = 0.032). No effect was observed among Black participants (*p* = 0.18). In other words, White participants who showed greater right lOFC activity in response to Black faces had better performance to Black faces, even though White and Black participants did not differ in overall performance (mean dprime scores). White participants with greater pro-white attitudes appear to contribute most to this association (Figure S6B; High attitudes r = 0.43, *p* = 0.036; Low attitudes r = 0.02, *p* = 0.92). No associations were observed between BOLD activity in the lOFC or participant race in predicting false-alarm RTs, providing evidence for the specificity of the above relationship to control regions (*p*s > 0.40 corrected).

## Discussion

Race implicitly biases our attitudes and beliefs about others (Perdue et al., 1990). However, the impact of race on explicit actions towards others is not fully understood. In the current study, we provide evidence that task-irrelevant race information contributes to diminished cognitive control, specifically to Black faces. Paralleling decreased cognitive control to Black faces, we observed greater activity in orbitofrontal and visual processing areas to Black faces. Exploratory brain-behavior analyses suggest that greater fusiform activity to Black faces is associated with faster impulsive errors for Black, but not for White individuals. In contrast, greater OFC activity was associated with better performance to Black faces in White participants when taking implicit racial associations into account. This brain-behavior dissociation between racial groups may provide preliminary evidence that superficially similar brain and behavioral responses to Black faces are differentially influenced by distinct underlying mechanisms in Black and White individuals (i.e., affiliative-related in-group vs. bias-related out-group interference).

The finding that individuals, regardless of their own race, exhibit diminished cognitive control to Black faces is consistent with our first hypothesis and previous research, which demonstrates increased attention and diminished cognitive control in response to Black cues (Bean et al., 2012; Correll et al., 2002, 2006; Donders et al., 2008; Kubota & Ito, 2007; Richeson et al., 2003, 2005; Trawalter et al., 2008). Our secondary analysis provides additional evidence of lower cognitive control performance to Black faces particularly when response inhibition is required. Previous work has largely examined behavior in response to Black faces in contexts where race information or racial stereotypes are salient (e.g., perceived threat). The current study provides evidence that race information can directly interfere with goal-directed behavior even when race is irrelevant to the task demands in a neutral context.

Previous research has implicated the role of the FFA in attentional bias, particularly to faces (Furey et al., 2006), and more recently in the encoding of in-group membership and race (Brosch et al., 2013; Golby et al., 2001). Whole-brain analyses revealed sensitivity to task-irrelevant stimulus race information in the bilateral FFA. Activity in the FFA also was sensitive to gender information (Figure S5B), the social category relevant to task performance. Sensitivity in the fusiform to both task-relevant gender cues and task-irrelevant race cues suggests a rapid and bottom-up attentional bias during visual processing of both task-relevant and task-irrelevant information. Even though participants were not instructed to direct their attention to race information in the current task, the diminished cognitive control performance paralleled by elevated FFA activity to Black faces by both racial groups may reflect interference by heightened attention to race information. Thus diminished cognitive control to Black faces may be modulated by enhanced attention to task-irrelevant race information over task-relevant gender information. This interpretation is consistent with studies showing heightened FFA activity to salient stimuli (e.g., angry faces; Kesler et al., 2001) and work showing sensitivity of the FFA to top-down attentional manipulations (Kaul et al., 2014; Van Bavel et al., 2011).

Although both Black and White participants showed diminished cognitive control to Black faces and showed similar recruitment of the FFA to Black faces, only Black participants showed an association between FFA activity and speed of impulsive errors  to Black faces. Specifically, Black individuals showed that greater FFA activity was associated with faster impulsive errors to Black faces, suggesting that Black individuals rapidly process in-group information that may interfere with task demands. This interpretation parallels previous research demonstrating both enhanced FFA activity to in-group faces (Golby et al., 2001; Lieberman et al., 2005) and sensitivity of the FFA to race information (Brosch et al., 2013; Golby et al., 2001). In-group affiliative processes offer one possible mechanism through which this relationship may be formed. Previous research on intergroup decision-making demonstrates that responding to in-group cues facilitates perceptual processing (Sui et al., 2012) and assessment of traits and behaviors (Coats et al., 2000; Katsumi & Dolcos, 2018; Smith & Henry, 2016), as measured by faster response times. Exploratory analyses suggesting that the association between fusiform activity and speed of impulsive errors is strongest among Black participants with implicit pro-black attitudes (Figure S6; Low attitudes *r* = −0.43, *p* = 0.038; High attitudes *r* = −0.14, *p* = 0.55) offers further support for this interpretation. Black participants also activated cortical midline structures (medial prefrontal cortex and precuneus) to a greater extent than White participants in response to Black versus White faces. The activation of these default network regions is consistent with prior work showing modulation of the default network by racial identification (Mathur et al., 2012). Thus, the observed association between BOLD response in the fusiform and rapid impulsive errors to Black faces among Black individuals may reflect affiliative-related enhanced attention to in-group information contributing to faster impulsive errors as a function of the participants’ own race. Enhanced fusiform activity to Black relative to White faces also was observed in White participants, although it did not correlate with speed of impulsive errors. Greater fusiform engagement to out-group relative to in-group faces diverges from the previously described studies of enhanced FFA activity to in-group faces (Golby et al., 2001; Lieberman et al., 2005; Reggev et al., 2020). However, this finding is consistent with the literature showing greater FFA activity when attention is focused on faces over competing stimulus information (Egner & Hirsch, 2005; Furey et al., 2006). As such, the fusiform activity to Black faces by White participants may reflect an attentional bias toward the more salient racial information over the task-relevant gender information.

The patterns of activation to Black versus White faces also revealed race sensitivity in the right lOFC, a neural structure implicated in stimulus-response conflict and response inhibition (Bryden & Roesch, 2015; Casey et al., 1997; Iversen & Mishkin, 1970; Jones & Mishkin, 1972). This finding is consistent with classic studies on the role of the OFC in response inhibition (Casey et al., 1997; Iversen & Mishkin, 1970; Jones & Mishkin, 1972). Evidence from single-unit recordings during an impulse control task suggests that the lOFC plays a role in dissociating between two perceptually-driven similar actions during response conflict rather than response inhibition (Bryden & Roesch, 2015). A positive relationship between right lOFC activity and cognitive control performance to Black faces was observed for White individuals only. Our results are consistent with previous findings in that elevated lOFC activity in response to Black faces was associated with better behavioral discrimination between the salient task-irrelevant race information and task-relevant gender information. Because Black and White participants did not differ in overall performance by the race of the stimulus (*p* = 0.90), the association between lOFC activity and better performance to Black faces by White participants may suggest that they experience more difficulty dissociating the task-relevant gender information from the salient yet task-irrelevant race information. It is important to note that this association is dependent on accounting for effects of implicit racial associations and appears to be driven predominantly by White participants with higher (more pro-white) implicit racial attitudes (Figure S6B). Although exploratory in nature, this pattern may provide evidence that inhibiting the interference of task-irrelevant race information is more effortful among White individuals who have the strongest negative associations with Black people.

It is important to underscore that the rapid and frequent cognitive control failures to Black faces were differentially related to brain activity in Black and White participants even though there were no differences in our index of cognitive control between racial groups. Together, our findings in the FFA and lOFC may reflect interference of task-irrelevant race information, which is differentially associated with cognitive control behavior to Black faces in Black and White individuals. The sample size in this mixed design precludes strong interpretations of our exploratory brain-behavior analyses, which yielded small effect sizes. However, there is an evolving focus on the implications of small effect sizes, where a single observation of a psychological process can have a consequential impact when accumulated repeatedly over time (Funder & Ozer, 2019; Götz et al., 2021). The possible associations suggested by our work between fusiform and orbitofrontal activity with behavioral performance in Black and White individuals provide guidance for future research studies.

Additional considerations of the task paradigm should be noted. The stimuli in the current paradigm were not equated for perceptions of threat, trustworthiness, or other trait judgements. However, it is important to note that Black faces are generally perceived as more threatening than White faces in neutral contexts (Kleider-Offutt et al., 2018; Shapiro et al., 2009), which therein lies the phenomenon we wished to capture in the current study. Second, the design of our paradigm relies on the rapid presentation of pictures of diverse faces. Although the paradigm is designed to isolate the behavioral and neural effects of each stimulus, it is possible that the effects from previous trials influenced the behavior and neural response in the current trials. Randomization of the order of stimulus presentations and jittered intertrial interval control for these effects across participants in part but may still contribute to the variance. Third, the near ceiling performance of hit rates regardless of stimulus race represents an additional limitation of the current study and raises uncertainty as to whether the task paradigm solicits sufficient cognitive control. It is important to note that because 70% of trials in the paradigm were go trials, we would expect participants to demonstrate a significant pre-potent bias to respond to trials. Indeed, response bias scores fall into the expected range for the parameters of the present go/no-go task (Young et al., 2018; Figure S2B). In addition, the intrasubject relationship between hit rates and false alarm rates (Figure S2A) provide evidence that false-alarm rates show significant variability despite uniformly high hit rates, suggesting that the present task was sufficiently difficult to engage cognitive control.

Theoretical and external validity considerations are also important when contextualizing the current findings within real-world behavior. While prior studies have typically focused on the effects of Black male stimuli on cognitive control performance (e.g., Brown et al., 2017; Correll et al., 2002), the current paradigm presents two salient social categories (race and gender). Previous research provides evidence that the categorization of gender can be biased by race information (Freeman et al., 2011; Johnson et al., 2012; Stolier & Freeman, 2016), suggesting that interactions between visual race and gender information may influence performance on the task. In the current study, however, we did not observe an interaction between stimulus race and stimulus gender. In addition, although we observe similar activity in the fusiform in whole brain race and gender contrasts across subjects (Figures 3 and S6, respectively), we observed lOFC activity exclusively when viewing Black versus White faces and not when viewing female versus male faces, suggesting that processing of race and gender information are relying in part on distinct neural processes. Finally, it is important to note that the task is a pared down approximation of an interracial social encounter. Social interactions are laden with continuous complex and dynamic decisions. As such, our study may minimize the effects and limit the implications of our findings for actual interracial encounter. Despite these considerations, this study takes an important step toward understanding cognitive control in response to racially diverse cues and, unlike much of the existing literature, allows us to parse stimulus race versus out-group effects by including an equal representation of Black and White participants and stimuli.

The present study examined how race influences behavior by embedding race information into a cognitive control task. We demonstrate that both Black and White individuals show diminished cognitive control in response to Black, compared to White, faces. Neuroimaging findings suggest that regions of the brain associated with face perception and stimulus-response conflict are preferentially engaged when responding to Black faces and that diverging processes between Black and White individuals may underlie superficially similar failures in cognitive control. The current study underscores the significant impact that race information has on our attention to and actions toward others. The inclusion of both Black and White participant groups in the present study allows for critical comparisons between racial groups in both brain and behavior and lays the foundation for further research to test hypotheses relevant to minority populations to inform future theoretical, empirical, and policy work. Prior interpretations of brain correlates of race and outgroup effects have been based on predominantly, or solely, White samples. Understanding the factors that underlie biased behaviors toward minoritized groups in racially and ethnically diverse samples is an imperative step toward inclusive research practices and identification of strategies to facilitate equitable behavior toward all individuals.

## Supplementary Information

ESM 1 (DOCX 915 kb)

## Footnotes

1 12.4% of total expected measurements for the linear regression predicting false-alarm RTs were missing due to no false alarms in specific conditions (e.g., White no-go, Black no-go trial). Five measurements were missing in White participants and 8 measurements were missing in Black participants.

2 To determine whether the relationship between false-alarm RTs and differential activity in the FFA was robust despite the possibility of high variability in false-alarm RT measurements (due to low false-alarm rates in some participants), we conducted the same regression analysis excluding participants who fell within the lowest quartile of either Black or White false-alarm rates. This resulted in a subsample in which subjects had at least two false-alarm trials for each stimulus race (N = 78; 39 Black). This analysis from a smaller sample revealed a similar but nonsignificant pattern of results as our original analysis with the full sample (B = −87.84, t(74) = *−1.72, p* = 0*.*091, *f*^*2*^ = 0.04, 95% CI [0.0, 0.18]). Simple slopes were not significant for Black (*p* = 0.18 or White (*p* = 0.28) subjects.

3 One participant was excluded with a BOLD response lower than 3 standard deviations below the mean.
