# Supplementary material for: Processing of Task-Irrelevant Race Information is Associated with Diminished Cognitive Control in Black and White Individuals
Source: Cogn Affect Behav Neurosci. 2021 May 3;21(3):625–38. doi: 10.3758/s13415-021-00896-8 (PMC8208919; doi:10.3758/s13415-021-00896-8)
Supplement: Supplementary file 1 — (DOCX 915 kb) [file 13415_2021_896_MOESM1_ESM.docx]

### Supplemental Material


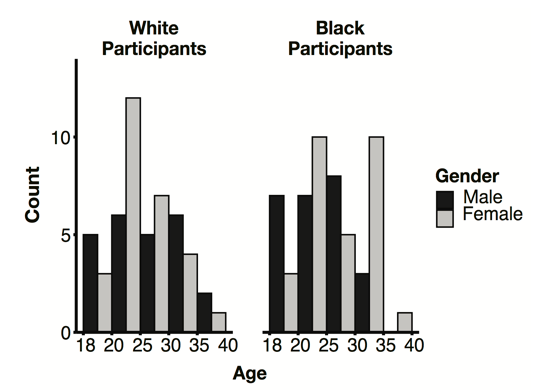


Figure S1. Histogram of participants with behavioral and imaging data by race, gender, and age range.


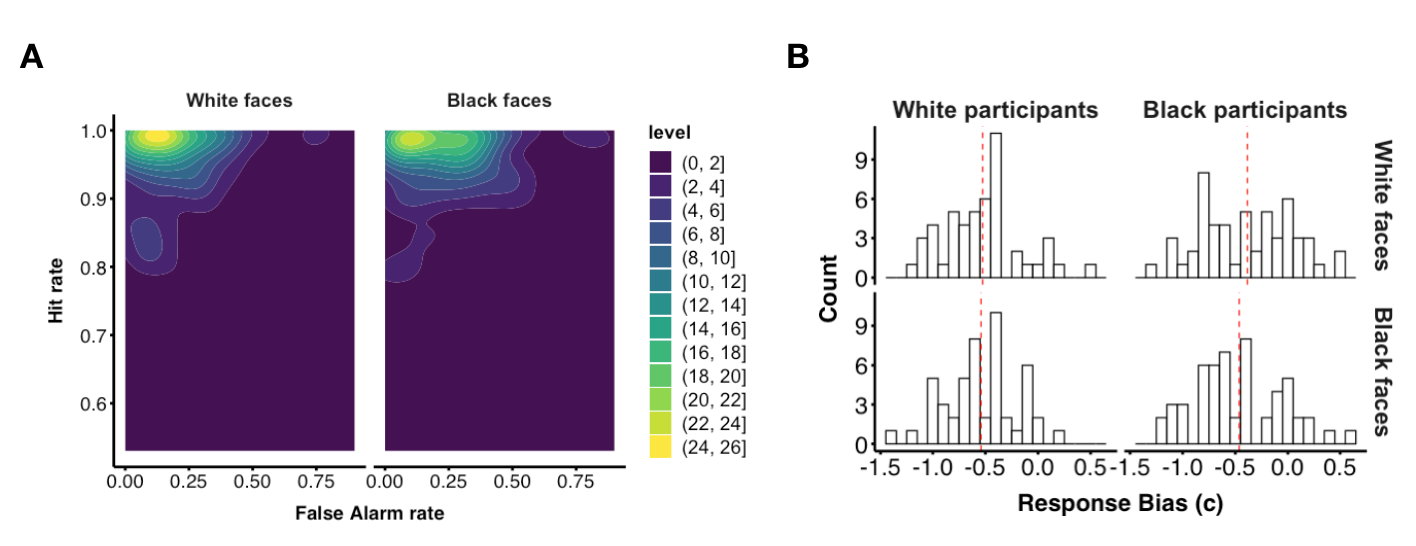


Figure S2. Intra-subject relationship between hit and false alarm rates. A) Density plots of hit rates and false alarm rates by stimulus race.  Warmer colors indicate a relatively greater number of participants who have hit and false alarm rates at the given values. B) Histogram of response bias scores (*c*) shows similar distribution across participant race and stimulus race. Red dotted lines represent mean response bias scores. Zero values indicate no response bias, and negative scores indicate a bias towards responding.


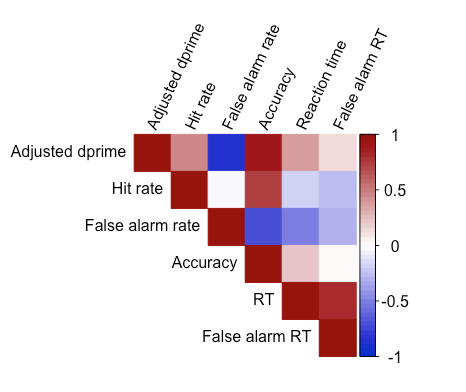


Figure S3. First order correlations between dependent variables in go/no-go task. Squares are colored to represent Pearson correlations as indicated on the scale (positive correlations in warm colors and negative correlations in cool colors).


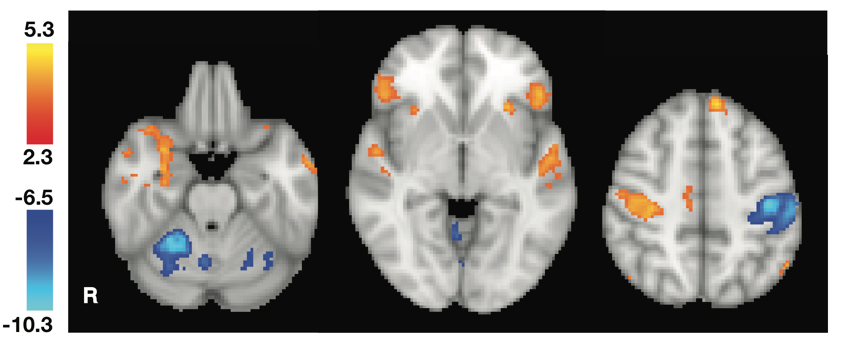


Figure S4. Participants activate canonical motor response circuitry during go trials (blue-light blue) and motor inhibition circuitry during no-go trials (red-yellow). Color scale depicts z-statistics.


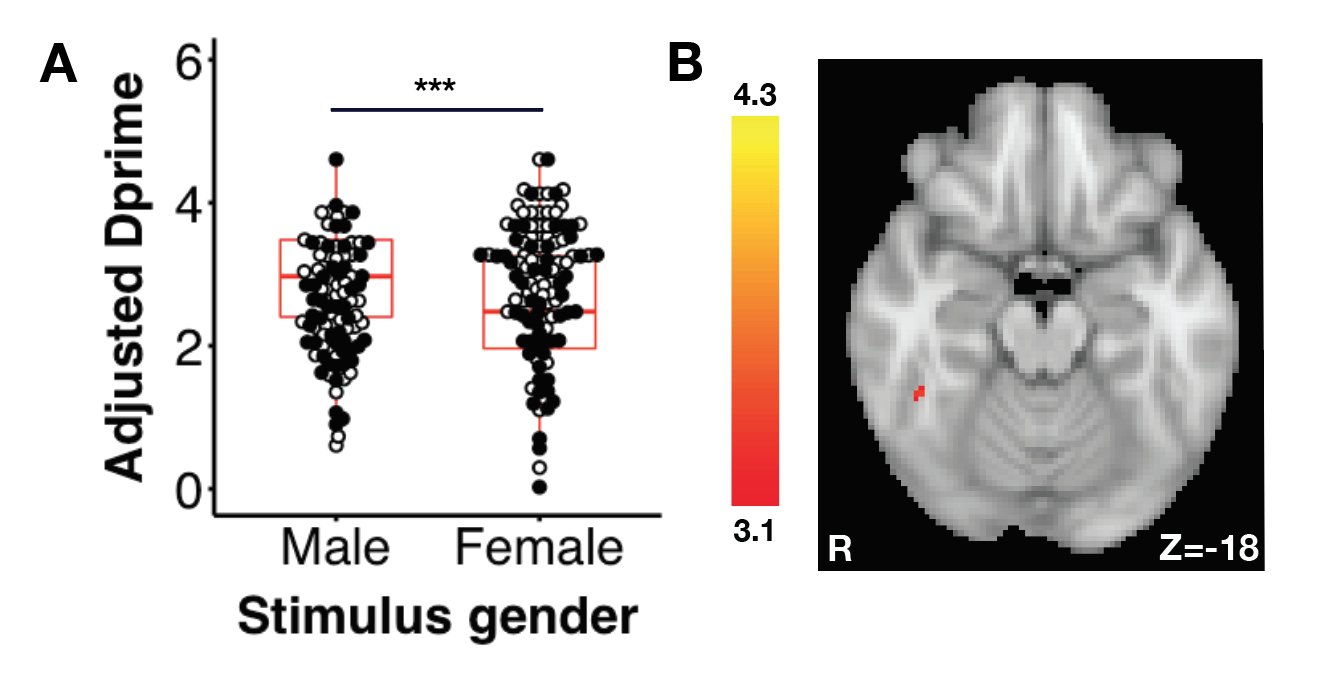


Figure S5. Stimulus gender associations with behavior and brain. A) Participants show lower dprime scores in response to Female versus Male faces. Boxplot represents the first and third quartile, median, and range of data points. Data points from male and female participants are represented by White and Black dots, respectively. B) Participants activate right fusiform in response to female versus male faces.


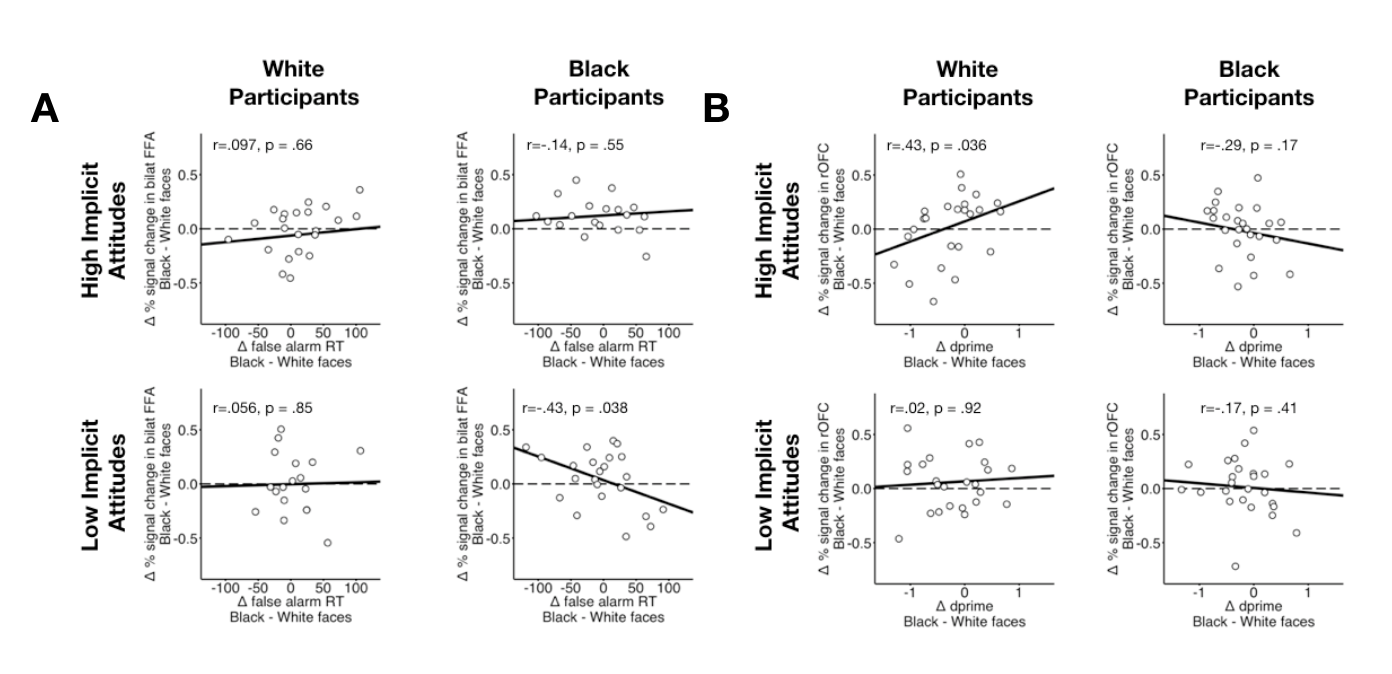


Figure S6. Associations between BOLD activity and task performance by implicit association scores and participant race in the A) bilateral fusiform and B) right lOFC. For visualization purposes, Black and White participants were categorized as having high (more pro-White) or low (more pro-Black) implicit attitudes using a median split in IAT scores for each participant group. Correlations between BOLD signal in the bilateral fusiform and rOFC and task performance were conducted separately for Black and White participants for high and low implicit racial associations.

Table S1. Linear mixed effects analysis predicting adjusted dprime scores

| \|  \| \| \| --- \| --- \| \|  \| *Dependent variable:* \| \|  \|  \| \|  \| Adjusted dprime (centered) \| \|  \| \| \| **Race of stimulus** \| **-0.184^**^** \| \|  \| **p = 0.007** \| \|  \|  \| \| **Gender of stimulus** \| **-0.467^***^** \| \|  \| **p < 0.001** \| \|  \|  \| \| Participant race \| -0.089 \| \|  \| p = 0.545 \| \|  \|  \| \| Participant gender \| 0.247 \| \|  \| p = 0.092 \| \|  \|  \| \| Race of stimulus x gender of stimulus \| -0.069 \| \|  \| p = 0.608 \| \|  \|  \| \| Participant race x race of stimulus \| 0.064 \| \|  \| p = 0.636 \| \|  \|  \| \| Constant \| -0.010 \| \|  \| p = 0.895 \| \|  \|  \| \|  \| \| \| Observations \| 420 \| \| Log Likelihood \| -520.523 \| \| \| Akaike Inf. Crit. \| 1,063.047 \| \| Bayesian Inf. Crit. \| 1,107.490 \| \|  \| \| \|  \| ^*^p<0.05; ^**^p<0.01; ^***^p<0.001 \| |  |
| --- | --- | --- | --- | --- | --- | --- | --- | --- | --- | --- | --- | --- | --- | --- | --- | --- | --- | --- | --- | --- | --- | --- | --- | --- | --- | --- | --- | --- | --- | --- | --- | --- | --- | --- | --- | --- | --- | --- | --- | --- | --- | --- | --- | --- | --- | --- | --- | --- | --- | --- | --- | --- | --- | --- | --- | --- | --- | --- | --- | --- | --- | --- | --- | --- | --- | --- | --- | --- |

*Note: All fixed vactors are represented with two-level deviation coding where White stimuli, male stimuli, White participants, and male participants are the reference groups.*

Table S2. Correct No go > Correct Go Activation Table

| Region | Peak coordinates (X, Y, Z) | Z_max_ statistic |
| --- | --- | --- |
| R inferior frontal gyrus, orbitofrontal, amygdala, hippocapus, middle temporal gyrus, inferior temporal gyrus, | -32, -42, -24 | 5.35 |
| L inferor frontal gyrus, orbiofrontal, insula | -56, +22, +22 | 4.81 |
| Medial prefrontal | -8, +42, +50 | 4.7 |
| R sensorimotor cortex | +52, -16, +58 | 4.87 |
| R parietal | +62, -50, +42 | 4.28 |
| L parietal | -58, -60, +38 | 4.22 |
| R sensorimotor | +6, -26, +60 | 4.62 |
| L superior temporal | -54, -38,+6 | 4.26 |
| L temporal pole | -42, -2, -32 | 4.39 |
| R temporal pole | +62, -10, -14 | 4.14 |

Table S3. Go > No – Go Contrast Activation Table

| Region | Peak coordinates (X, Y, Z) | Z_max_ statistic |
| --- | --- | --- |
| L motor, sensorimotor cortex, parietal operculum | -40, -20, +50 | 10.3 |
| R cerebellum | +16, -50, -20 | 10.2 |
| R cerebellum | +20, -60, -46 | 9.21 |
| L central operculum | -46, -4, +8 | 9.14 |
| L cerebellum | -18, -70, -18 | 8.31 |
| L motor cortex | -56, 4, 24 | 7.83 |

Table S4. Black faces > White faces Activation Table

| Region | Peak coordinates (X, Y, Z) | Z_max_ statistic |
| --- | --- | --- |
| R fusiform | +42, -54, -14 | 4.25 |
| R orbitofrontal | +32, +20, +4 | 4.08 |
| L fusiform | -32, -42, -24 | 3.86 |
|  |  |  |

Table S5. Stimulus Race by Participant Race Interaction: Black > White Participants in Black > White Faces Activation Table

| Region | Peak coordinates (X, Y, Z) | Z_max_ statistic |
| --- | --- | --- |
| R parietal | +50, -70, +28 | 4.72 |
| L parietal | -30, -84, +40 | 4.30 |
| Ventromedial PFC | -2, +68, -6 | 4.06 |
| Precuneus | +8, -60, +18 | 3.77 |

Stimulus Gender Imaging Analysis

Eight first-level regressors were created for each participant: Male Correct Go trials, Female Go Correct trials, Male Correct No-go trials, Female Correct No-go trials, Male Incorrect Go trials, Female Go Incorrect trials, Male Incorrect No-go trials, and Female Incorrect No-go trials. Each trial was modeled for 500 milliseconds with a double-gamma hemodynamic response function. All subsequent analyses steps mirrored stimulus race FSL analyses. Group level analysis included between-subjects factors of participant race, participant gender, and scanning site.
